# Meal replacement: calming the hot-state brain network of appetite

**DOI:** 10.3389/fpsyg.2014.00249

**Published:** 2014-03-25

**Authors:** Brielle M. Paolini, Paul J. Laurienti, James Norris, W. Jack Rejeski

**Affiliations:** ^1^Department of Radiology, Wake Forest University School of MedicineWinston-Salem, NC, USA; ^2^Translational Science Center, Wake Forest UniversityWinston-Salem, NC, USA; ^3^Department of Mathematics, Wake Forest UniversityWinston-Salem, NC, USA; ^4^Department of Health and Exercise Science, Wake Forest UniversityWinston-Salem, NC, USA; ^5^Department of Geriatric Medicine, Wake Forest UniversityWinston-Salem, NC, USA

**Keywords:** meal replacement, craving, eating behavior, obesity, brain networks, graph-theory

## Abstract

There is a growing awareness in the field of neuroscience that the self-regulation of eating behavior is driven by complex networks within the brain. These networks may be vulnerable to “hot states” which people can move into and out of dynamically throughout the course of a day as a function of changes in affect or visceral cues. The goal of the current study was to identify and determine differences in the Hot-state Brain Network of Appetite (HBN-A) that exists after a brief period of food restraint followed either by the consumption of a meal replacement (MR) or water. Fourteen overweight/obese adults came to our laboratory on two different occasions. Both times they consumed a controlled breakfast meal and then were restricted from eating for 2.5 h prior to an MRI scan. On one visit, they consumed a meal replacement (MR) liquid meal after this period of food restriction; on the other visit they consumed an equal amount of water. After these manipulations, the participants underwent a resting fMRI scan. Our first study aim employed an exploratory, data-driven approach to identify hubs relevant to the HBN-A. Using data from the water condition, five regions were found to be the hubs or nodes of the HBN-A: insula, anterior cingulated cortex, the superior temporal pole, the amygdala, and the hippocampus. We then demonstrated that the consumption of a liquid MR dampened interconnectivity between the nodes of the HBN-A as compared to water. Importantly and consistent with these network data, the consumption of a MR beverage also lowered state cravings and hunger.

## Introduction

There is a growing awareness in the field of neuroscience that the self-regulation of eating behavior is driven by complex networks within the brain that control “liking” and “wanting” of food. In extensive research, Berridge et al. (2010) have shown that “liking” represents the hedonic facet of this process whereas “wanting” refers to incentive salience motivation. The authors emphasize that “wanting” can be motivational even when hedonic “liking” does not arise. Although it is not possible to discern “liking” from “wanting” in most experimental paradigms, there is evidence that the desire to consume food is based upon activity in brain networks that vary as a function of individual differences (Rejeski et al., 2012), environmental stimuli (Stoeckel et al., 2009; Bullins et al., 2013; Kullmann et al., 2013), and homeostatic drive (Berthoud, 2012). In line with Loewenstein (2005), people can move in and out of “hot states” dynamically throughout the course of a day as a function of changes in affect or visceral cues; thus, the desire for food or the preoccupation with it is disproportionately higher in hot than cold states (see also Kavanaugh, 2005). The goal of the current study is to identify and determine differences in the hot-state brain network of appetite (HBN-A) that exists in a resting state after a brief period of food restraint followed either by the consumption of a meal replacement (MR) or water.

The study design we employed involved having participants (a) come to our laboratory on two occasions so that we could feed them a controlled morning meal, (b) insure that they did not eat for 2.5 h, (c) deliver on a randomized schedule either water or a MR, and (d) then have them participate in resting fMRI scans. Thus, we were interested in whether there was an identifiable HBN-A in a resting state following a brief period of food restraint and, whether MR calmed this network as compared to water. In identifying the HBN-A, we were guided by both existing research and an empirically-driven process. Specifically, a recent review by Stice and colleagues has summarized the integrative signaling of the brain reward system, including both the homeostatic and hedonic feeding systems (Lowe and Butryn, 2007; Stice et al., 2013). Much work has been done in this field by other investigators as well (Berridge et al., 2010; Kringelbach et al., 2012); however, the review by Stice et al. (2013) is one of the most comprehensive in identifying areas at all levels of the brain, from the subcortical to the neocortex, that are involved in eating behavior. Based upon this review and other published work (Bechara et al., 2000; Tracy et al., 2001; Kringelbach, 2004; Olson et al., 2007; Stoeckel et al., 2008; Pessoa, 2010; Carnell et al., 2012; Kringelbach et al., 2012; Paolini et al., 2012; Rejeski et al., 2012), we anticipated that the HBN-A would consist of at least 4 primary regions: the insula, hippocampus, amygdala, and anterior cingulate cortex. Due to limitations of fMRI technology, we did not anticipate detecting small, deep brain structures such as the parabrachial nucleus or the ventral tegmental area identified by previous investigators (Kringelbach et al., 2012; Stice et al., 2013). Similarly, because our study involved resting state without active processing of food cues, we did not expect involvement of the orbitofrontal cortex (OPF) (Kringelbach et al., 2012).

Because we are unaware of existing fMRI research on this topic using graph-theory-based methodology, the first phase of our analysis was designed to qualitatively evaluate and empirically confirm the network hubs of relevance to the HBN-A. To ensure high sensitivity in this phase, we used a *p* value of 0.10 to identify network hubs. Once the structure of the HBN-A was established, we then examined whether the consumption of a MR altered connectivity in this network as compared to water. This second phase, which was the primary aim of the study, employed a per comparison error rate of *p* = 0.05. Based upon clinical research which has shown that MR products are effective in curbing appetite and in promoting weight loss (Rothacker et al., 2001; Heymsfield et al., 2003; Annunziato et al., 2009; Frestedt et al., 2012), we hypothesized that MR would decrease connectivity within the HBN-A when compared to water and that this effect would be evident for both direct and indirect connections within the HBN-A. We also evaluated state craving and hunger and expected both to be higher in the water than MR condition.

## Methods

### Participants

A sample (*n* = 14) of older, overweight and obese (BMI ≥ 28 kg/m^2^ but ≤ 40 kg/m^2^) adults was recruited from Forsyth County, NC. All participants were between the ages of 50 and 79 and lived independently. The sample included an equal number of men and women. Each participant completed a phone screen, an in-person screening visit, and two 5-h experimental sessions at Wake Forest School of Medicine, receiving a maximum of $225 for completing all three visits to compensate for their time.

### Prescreening and lost to follow-up

A telephone screen was administered to interested individuals to determine their qualifications as potential participants. Exclusion criteria included (1) having a BMI outside our established range, (2) the presence of a systemic uncontrolled disease or psychiatric illness, (3) a binge eating disorder, (4) high alcoholic intake (more than 3 drinks per day), (5) the inability to safely undergo magnetic resonance imaging due to claustrophobia or to the presence of implanted magnetic objects/devices, (6) currently undergoing treatment for cancer, (7) active participation in another research study that might interfere with either the study's procedures or objectives, (8) need for assistance while walking, (9) being unable to read or speak English, or (10) the inability to correct eyesight to at least 20/40 in the scanner to complete the required tasks. Over 125 people were contacted by phone about the study, of these 22 were brought in for in-person screening visits, 7 participants did not complete the study due to various reasons: withdrawal due to claustrophobia and poor vision in the scanner. One participant was excluded from the data analyses due to poor quality of brain images during one functional scan that could not be corrected using computer software.

### Measures

#### Food craving questionnaire (the FCQ_state_)

The state version of the FCQ was used to measure food cravings and to obtain a measure of hunger as a manipulation check (Cepeda-Benito et al., 2000). The craving measure consists of 15 items that target preferred foods using a 5-point scale (1 = strongly disagree; 5 = strongly agree) with the mid-point being anchored by the label neutral; thus, total scores can range from 15 to 75. In our own work, we have found the FCQ state to be very sensitive to food restraint (Rejeski et al., 2010). The 15-items are averaged for a total score. In addition, three items from the FCQ can be used to derive an index of hunger. The Cronbach alpha internal consistency reliabilities in this study for both scales were excellent, ≥0.90.

#### The interview for the diagnosis of eating disorders (IDED-IV)

The semi-structured interview described by Kutlesic et al. (1998) was employed to exclude any potential participants that might have a binge-eating disorder as defined by the DSM-IV criteria Dr. Williamson, an investigator involved in the development of the IDED-IV, provided the training on how to screen for Binge Eating.

### In-person screening and assessments

An in-person screening visit was completed to obtain an informed consent, collect biometric data, assess current states of physical activity and possible dieting, and to screen for binge eating disorders. The IDED-IV was used to identify and exclude people with possible eating disorders. Eligible individuals were scheduled for two imaging visits 7–10 days apart. If necessary, participants were fitted for MRI-safe corrective lenses to be used in the scanner during computer tasks.

### Experimental protocol for the scanning visits

Participants completed two 5-h visits beginning in the early morning around 8:00 a.m. Participants were asked to arrive in a fasting state, having not eaten breakfast or consumed anything other than water. During each visit, participants ate a prepared breakfast containing 350 calories for females and 450 calories for males. The meals were designed by a staff nutritionist to provide a heart healthy balance of macronutrients containing approximately 25% fat, 15% protein, and 60% carbohydrates. Participants were allowed to choose macronutrients from a menu. Following the consumption of at least 75% of their breakfast, participants completed a baseline FCQ_state_. The participants then fasted for 2.5 h under the supervision of research center nursing staff.

Approximately 45 min before the imaging procedure, the research staff then administered the MRI safety form and led each participant in a practice session of the tasks to be completed during the fMRI. About 30 min before the scan time, participants either consumed a can of the Nestle MR beverage BOOST® (short term energy surfeit containing 240 calories, vanilla flavor) or an equivalent volume of water. They then completed a second round of the FCQ_state_. The assignment of the MR and water condition was randomized.

### Resting state scanning tasks

Participants wore goggles (Resonance Technology, www.mrivideo.com) in the scanner that were directly interfaced with a computer screen. The MRI consisted of a resting-state session where individuals viewed a cross on the computer screen interfaced with their goggles for a period of 5 min.

### Scanning protocol

All scans were performed on a 1.5 GTE scanner using an 8 channel neurovascular head coil (GE Medical Systems, Milwaukee, WI, USA) and included anatomic imaging, perfusion, and one resting state fMRI. All fMRI data was used to evaluate differences in brain networks between each individual's MR and water treatment condition.

Functional images for the network analyzes measured changes in the T2^*^-relaxation rate that accompany changes in blood oxygenation. The T2^*^ signal is sensitive to changes in blood oxygen content. As brain activity changes, the oxygen content of the blood in the same area also changes. Thus, the T2^*^ signal is an indirect measure of changes in neural activity (Ogawa et al., 1990). Functional imaging was performed using multi-slice gradient EPI (*TR* = 2000 ms; *TE* = 40 ms; field of view = 24 cm (frequency) × 15 cm (phase); matrix size = 3.75 × 3.75 × 5 mm).

### Imaging processing and network analyses

In preparation for generating brain networks, all scanning images were realigned and normalized to standard space using FSL (Smith et al., 2004). The time courses were extracted for each voxel in gray matter based on the Automated Anatomical Labeling atlas (Tzourio-Mazoyer et al., 2002) and band-pass filtered to remove signals outside the 0.009–0.08 Hz range (Biswal et al., 1995). To account for physiological noise, mean white matter, CSF, and motion correction parameters were regressed from the filtered time series. This regression procedure removes signal fluctuations that are unlikely to be from neuronal activity (Fox et al., 2005). A correlation matrix was then created by computing Pearson correlations between all possible pairs of voxels (~21,000 voxels). This produced a 21,000 × 21,000 matrix which each cell (ij) representing the correlation coefficient between nodes i and j. A threshold was then applied to the correlation matrix and all cells that surpassed this threshold were assigned a value of 1 while remaining cells were assigned a value of zero. The threshold was defined so that the relationship between the number of nodes and average number of connections at each node was consistent across subjects to produce an adjacency matrix. Specifically, the relationship *S* = log(*N*)/log−(*K*) was the same across subjects as described above (Hayasaka and Laurienti, 2010). The threshold *S* = 2.5 was used for this paper. This resulted in networks with connection densities meeting expected values based on the size of the networks (Laurienti et al., 2011). All remaining analyses were completed using the binary 21,000 × 21,000 adjacency matrix.

To define hubs-of-interest, we generated degree (K) maps for each individual. Degree is the number of functional connections linked to a node. For each individual, we generate a degree (K) map which gives a degree value (i.e., number of connections) for each voxel in the network. In order to compare regions-of-interest (ROIs) between the two conditions, we generated the average number of connections for each ROI for each individual. This allowed us to run a paired *t*-test to test the difference in degree or number of connections for each ROI between the MR and water conditions. The same ROIs defined below were used to evaluate average degree for each individual.

**Figure 1:**
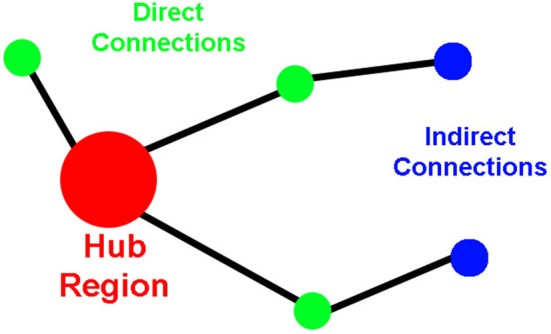
**Cartoon demonstrating the properties of the direct and the indirect connections to network hubs**.

To assess network organization, first order (direct) and second order (indirect) connection analyses were performed for ROIs that qualified as hubs-of-interest. First order connections are the immediate network neighbors of the hubs identified because they share direct connections. In this paper, we calculate the direct connections as the number of direct connections between two ROIs (i.e., the insula and the amygdala). Second order connections are areas that have direct connections to the immediate neighbors of the hubs (Figure 1). Indirect connections between two ROIs can be asymmetric because the ROIs may or may not share the same number of connections to those neighbors. This asymmetry for indirect connections between two hubs does not reflect a difference in directionality of information flow; instead, it reflects a difference in the complexity of the connections between the two ROIs. For instance, a large number of indirect connections from ROI-A to ROI-B indicates a large number of connections from ROI-A's neighbors to ROI-B; whereas, a small number of indirect connections from ROI-A to ROI-B indicates that there are a small number of connections from ROI-B's neighbors to ROI-A.

For our analysis, the following ROIs were used: insula, defined by the Automated Anatomic Labeling (AAL) atlas; amygdalae, defined by the AAL atlas; the superior temporal pole, defined by the AAL atlas; anterior cingulate cortex, defined as a sphere with a radius of 10 mm with the MNI coordinates (*x* = 4, *y* = 38, *z* = 0); the right hippocampus, defined as a sphere with a radius of 6 mm and the MNI coordinates (*x* = 21, *y* = −7, *z* = −18) based on the region with the highest degree in the degree map. The ACC and the right hippocampus ROIs used were determined by drawing spheres over the region using the WFU pick-atlas software (Tzourio-Mazoyer et al., 2002; Maldjian et al., 2003). For each ROI created with the exception of the hippocampus, both the left and right corresponding regions were included. The ROI used for the hippocampus was unilateral because the right side was the only side that differed between MR and water conditions.

The ROIs generated in pick-atlas software are at a 2 × 2 × 2 mm resolution. All ROIs were resliced to 4 × 4 × 5 mm, the resolution of the imaging data, for this analysis. Standard reslicing practices combine a subset of original voxels into a new larger sized voxel. This process causes adjacent ROIs to overlap and to share common voxels. To correct for this issue, every resliced voxel is assigned to a single ROI. This assignment is based on the most frequent assignment of the original 2 × 2 × 2 mm voxels in the AAL atlas.

### Analytical strategy and statistical analyses

In this paper, resting brain network data collected during the water condition was used to provide empirical support for the existence of a hot-state brain network for appetite (HBN-A). We relied on both extant research and an empirical approach to locate “hubs of interest” in the HBN-A during our first-stage, exploratory analysis. Thus, we used a data-drive approach to the identification of our hubs. The hubs were then used as seeds for further analysis of connectivity within the network, which was our primary outcome. It is important to note that we used node degree to inform our connectivity analyses, and the connectivity analyses to inform our identification of hubs. We defined hubs-of-interest as regions-of-interest (ROIs) that had a larger degree (i.e., greater number of connections) in the water than MR condition. For our first-stage, exploratory analysis of hubs of interest, we set the alpha level for hubs-of-interest at *p* < 0.10 which allowed us to capture ROIs that may have important connectivity profiles despite not reaching a conventional level of significance using the degree metric.

In addition, a second phase for validating the conceptual import of each hub was to examine first order (direct) and second order (indirect) connections between the hubs-of-interest. To be included in the HBN-A, hubs not only had to pass the first level of screening based on degree, but also had to show evidence of either first or second order connectivity to one of the other hubs-of-interest. If the hub met these two criteria, it was then considered a node in the HBN-A. Once the nodes of the HBN-A were established, we then compared connectivity in this network for the water condition vs. the MR treatment condition. It is important to note that all hypothesis tests conducted were based on paired *t*-tests and a 0.05 pairwise level of significance.

We used a mixed model ANCOVA to test the effect of the MR manipulation on the food craving questionnaire. In this analysis, we controlled for the assessment taken at the time of the post-breakfast feeding as well as the random subject effect. The outcome of interest was the craving responses taken just prior to conducting the fMRI scans.

## Results

Participants (7 males and 7 females) had a mean (*SD*) age of 71.35 (4.92) years with a Body Mass Index of 30.43 (2.09). Two of our participants were African American and the remaining 12 were Caucasian. The leading comorbidities included arthritis (*n* = 8) and hypertension (*n* = 4); these health conditions were followed by cancer (*n* = 2) and cardiovascular disease (*n* = 1).

The efficacy of the MR manipulation was supported by data showing that ratings of hunger from the food craving inventory were higher on the day that participants consumed water prior to the scanning procedure, mean (SE) = 9.00 (0.67), as compared to the day they received a MR, mean (SE) = 6.78 (0.75); *t* = 2.14 (13), *p* = 0.05.

### Craving

As discussed above, a mixed-model ANCOVA was employed to examine how participants' level of state craving was influenced by the two treatment conditions (MR or water). Analysis of the FCQ_state_ measure produced a significant main effect for the MR manipulation [*F*_(1, 12)_ = 6.23, *p* = 0.028]. Cravings were significantly higher in the water than the MR condition, respectively: LS means (SE) = 41.74 (2.41) vs. 33.62 (2.41).

### Network analyses

**Figure 2:**
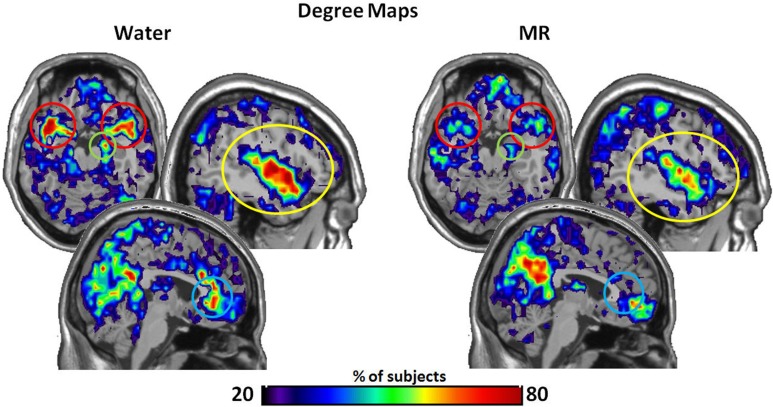
**The maps demonstrate brain areas found to consistently have high degree (i.e. number of connections)**. For each subject, the voxels with degree values in the top 20% were identified. The maps shown here represent the overlap of these voxels across subjects in each condition. The consistency of overlap between conditions is indicated by the color bar which represents the percentage of individuals for which each voxel was among the top 20%. On the top left are axial slices (MNI *z* = 54) through the superior temporal pole and amygdala (red circle) and the right hippocampus (green circle). On the top right are sagittal slices (MNI *x* = 139) through the insula (yellow circle). Finally the images on the bottom are a sagittal slices (MNI *x* = 95) through the anterior cingulate (blue circle). Figures 3–7 are shown with these same slices. *The figure highlights that the superior temporal pole, right hippocampus, the ACC, and the insula have greater connectivity in the water condition than in the MR condition during resting state.*

Our initial analysis examined which ROIs served as hubs-of-interest for the hot-state network. This step involved a qualitative comparison of the resting brain networks in the water and MR treatment conditions that was guided by evidence from existing research. We then conducted statistical tests to empirically validate the qualitative findings. Using this method, five hubs met the criteria and were included as nodes of the HBN-A: ACC (*p* = 0.053), the right hippocampus (*p* = 0.030), superior temporal pole (*p* = 0.029), insula (*p* = 0.030), and amygdala (*p* = 0.079). Table 1 provides results for the between condition statistical analyses of these hubs; whereas, Figure 2 shows the location of the high-degree nodes that were spatially consistent across individuals.

**Table 1:** **Summary of hubs of the HBN-A**.

**Degree profile**	**Water [Mean, (SE)]**	**MR [Mean, (SE)]**	***p*-value**
ACC	78.000 (10.689)	50.888 (6.879)	0.053
Hippocampus	127.152 (24.982)	61.800 (7.900)	0.030^*^
STP	120.051 (20.866)	76.790 (8.820)	0.029^*^
Insula	73.127 (8.321)	49.703 (5.412)	0.030^*^
Amygdala	48.42 (7.103)	34.485 (6.380)	0.079
Precuneus	83.348 (12.171)	133.551 (24.868)	0.050

STP, superior temporal pole; ACC, Anterior Cingulate Cortex;

* , significance.

Within Figure 2 it is also important to note that the precuneus had higher degree in the MR than the water condition (*p* = 0.05). However, in defining the HBN-A, we were interested in ROIs that were hubs in the water condition; these ROIs did not include the precuneus. Moreover, the precuneus did not have a greater number of connections in the water condition compared to the MR condition or to any of the other hubs-of-interest in the HBN-A.

**Figure 3:**
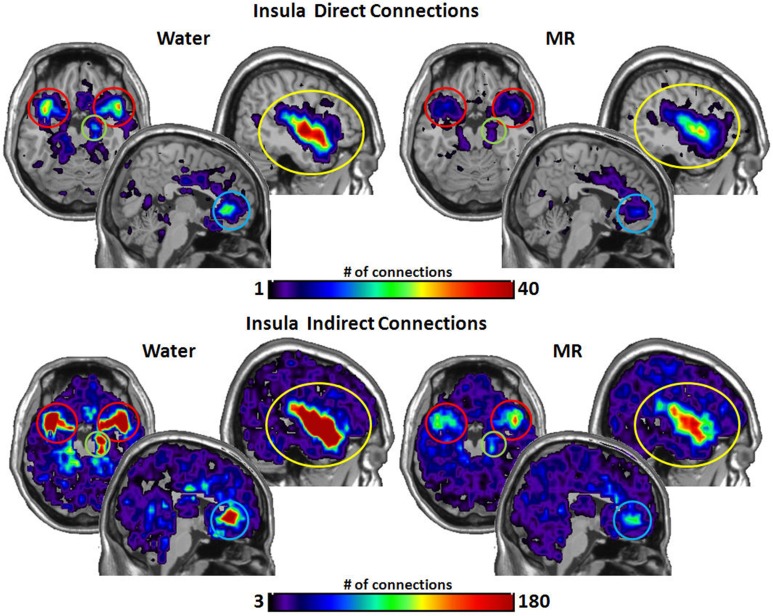
**These maps show the total number of connections from the insula during resting state averaged across individuals**. The brain images on the top demonstrate the insula's average number of direct connections with other brain regions. *These images demonstrate that the insula has a greater number of direct connections with the ACC, STP, Amygdala, and the hippocampus during the water condition vs. the MR condition.* The brain images on the bottom demonstrate the total number of indirect connections from the insula averaged across participants. *These images demonstrate that during the water condition, the insula has a greater number of indirect connections with the ACC, STP, amygdala, and hippocampus than during the MR condition.*

**Figure 4:**
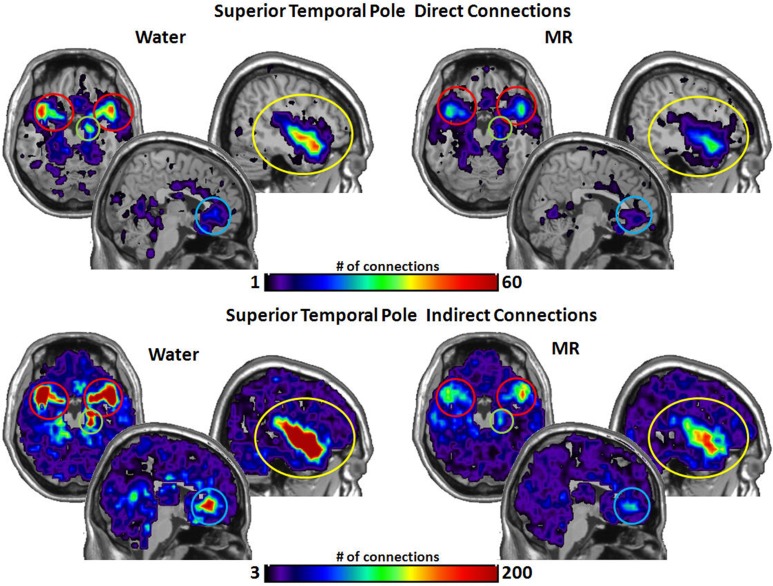
**The brain images on the top demonstrate the total number of direct connections from the superior temporal pole (STP) averaged across participants**. *These images demonstrate that the STP has a greater number of direct connections with the insula and the hippocampus during the water than in the MR condition.* The difference in direct connections with the ACC and the amygdala did not reach significance (*p* = 0.059 and *p* = 0.052, respectively). The brain images on the bottom demonstrate the total number of indirect connections from the STP averaged across individuals. These images demonstrate that during the water condition the STP has a greater number of indirect connections to the insula, ACC, and the hippocampus than during the MR condition. The indirect connections to the insula were marginally significant (*p* = 0.051).

**Figure 5:**
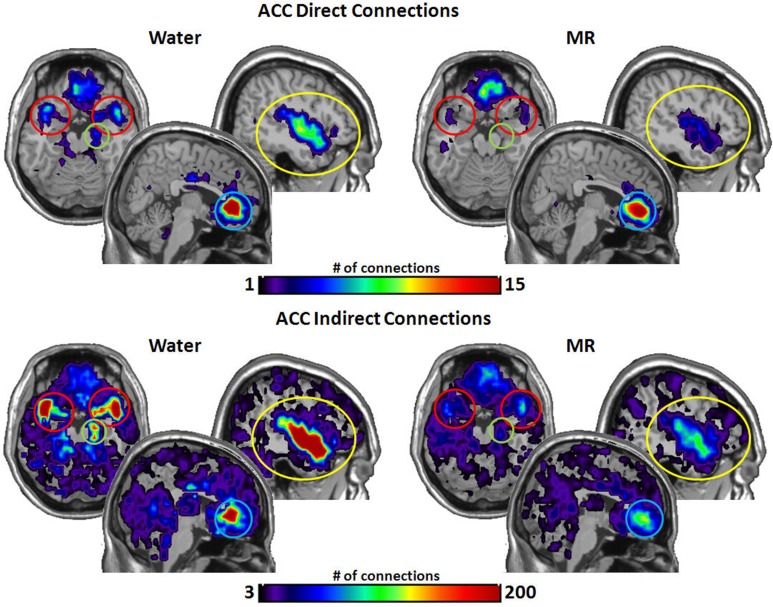
**The brain images on the top demonstrate the total number of direct connections from the anterior cingulated cortex (ACC) averaged across participants**. *These images demonstrate that the ACC has a greater number of direct connections to the insula during the water than in the MR condition.* It appears that the ACC also has greater direct connections with the STP in the water condition; however, this trend did not reach statistical significance (0.059). The brain images on the bottom demonstrate the total number of indirect connections from the ACC averaged across individuals. These images demonstrate that during the water condition the ACC has a greater number of indirect connections to the insula, ACC, the amygdala and the hippocampus than during the MR condition.

**Figure 6:**
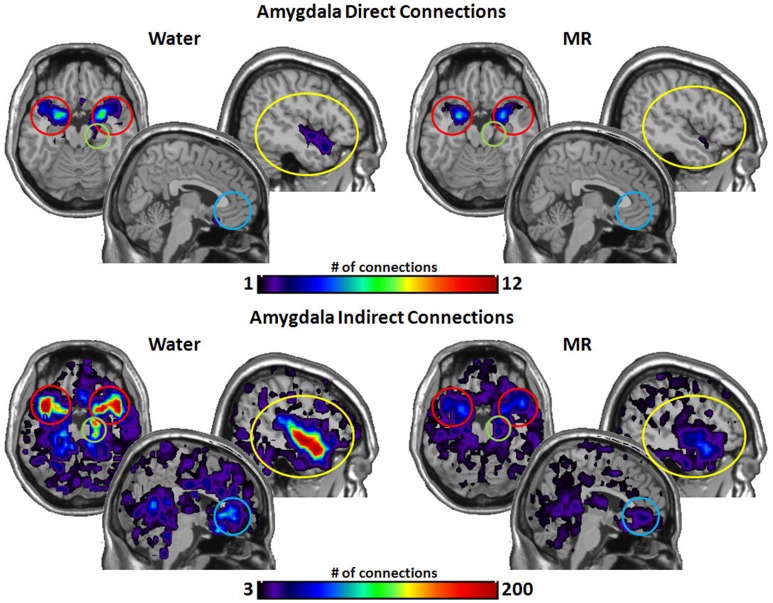
**The brain images on the top demonstrate the total number of direct connections from the amygdalae averaged across participants**. *These top images demonstrate that the amygdala has a greater number of direct connections with the insula during the water than in the MR condition.* The brain images on the bottom demonstrate the total number of indirect connections from the amygdalae averaged across participants. *These images demonstrate that the amygdala has a greater number of indirect connections to the insula, the hippocampus, and the STP during the water condition than during the MR condition.* Despite visually appearing to have a greater number of connections to the ACC, this trend did not reach statistical significance (*p* = 0.053).

Figures 3–6 illustrate the number of direct (upper panel in each figure) and indirect (lower panel in each figure) connections between each of the five nodes identified in Figure 2. Supporting data from statistical tests can be found in Tables 2, 3. In each case, the number of connections is always higher in the water condition as compared to the MR condition. Also, a large number of direct and indirect connections can be observed within each seed ROI; however, this would be expected. For defining the HBN-A, we were interested in the interconnections from each ROI to every other ROI in the HBN-A.

**Table 2:** **Summary of direct connections between nodes of the HBN-A**.

**Total number of direct connections**	**Water [Mean, (SE)]**	**MR [Mean, (SE)]**	***p*-value**
Insula–Amygdala	132.371 (26.765)	77.429 (18.798)	0.045^*^
Insula–Hippocampus	209.143 (60.357)	51.286 (12.501)	0.029^*^
Insula–STP	2610.571 (485.806)	1278.000 (197.122)	0.018^*^
Insula–ACC	707.571 (140.501)	285.214 (87.067)	0.021^*^
STP–Amygdala	345.857 (77.915)	178.143 (45.532)	0.052
STP–ACC	593.786 (173.683)	234.143 (83.338)	0.059
STP–Hippocampus	529.000 (105.903)	220.643 (43.129)	0.016^*^
ACC–Hippocampus	57.000 (25.035)	10.214 (3.598)	0.091
ACC–Amygdala	18.357 (5.990)	9.071 (3.656)	0.180
Amygdala–Hippocampus	59.286 (16.611)	27.929 (9.765)	0.062

* , 0.05.

**Table 3:** **Summary of indirect connections between nodes of the HBN-A**.

**Total number of indirect connections**	**Water [Mean, (SE)]**	**MR [Mean, (SE)]**	***p*-value**
Insula–Amygdala	2107.286 (399.922)	1257.357 (306.347)	0.050^*^
Insula–Hippocampus	3332.143 (765.529)	1308.143 (222.055)	0.030^*^
Insula–STP	31331.643 (6325.345)	18182.214 (2618.435)	0.032^*^
Insula–ACC	8066.429 (1419.632)	3599.714 (796.690)	0.024^*^
STP–Amygdala	2352.071 (403.201)	1479.786 (342.118)	0.051
STP–ACC	7882.286 (1457.577)	3428.429 (585.329)	0.017^*^
STP–Hippocampus	3646.000 (752.764)	1599.000 (249.125)	0.027^*^
STP–Insula	26656.071 (3678.668)	15925.429 (2047.362)	0.021^*^
ACC–Insula	21480.000 (3781.479)	8227.286 (2078.500)	0.009^*^
ACC–Hippocampus	2429.143 (772.684)	569.857 (187.532)	0.035^*^
ACC–Amygdala	1180.857 (375.519)	346.571 (96.477)	0.041^*^
ACC–STP	24000.214 (6547.866)	8839.000 (2725.875)	0.024^*^
Amygdala–STP	21729.000 (5951.026)	6780.000 (1412.635)	0.022^*^
Amygdala–ACC	3267.643 (1089.861)	866.571 (247.474)	0.053
Amygdala–Hippocampus	2613.071 (624.028)	724.500 (192.236)	0.015^*^
Amygdala–Insula	12309.071 (2931.547)	3983.714 (978.878)	0.016^*^
Hippocampus–Amygdala	1615.429 (431.436)	775.786 (261.550)	0.068
Hippocampus–ACC	4155.786 (1491.771)	793.214 (242.229)	0.048^*^
Hippocampus–STP	23542.000 (6573.370)	8037.857 (2161.360)	0.025^*^
Hippocampus–Insula	14417.071 (3946.902)	3269.214 (795.160)	0.022^*^

* , 0.05.

Inspection of the data in Figure 3 shows that the insula had significantly more direct connections with the superior temporal pole (STP) (red circle, *p* = 0.018), to the anterior cingulate cortex (ACC) (blue circle, *p* = 0.021), the hippocampus (green circle, *p* = 0.029), and the amygdala (red circle, *p* = 0.045) in the water than the MR condition. The insula also exhibited greater indirect connections to the same four nodes within the HBN-A during the water condition: the STP (red circle, *p* = 0.032), the ACC (blue circle, *p* = 0.024), hippocampus (green circle, *p* = 0.030) and the amygdala (red circle, *p* = 0.050).

As shown in Figure 4, the STP had significantly more direct connections with the insula (*p* = 0.018) and with the hippocampus (*p* = 0.016) in the water than MR condition. Although the STP appears to have had a greater number of direct connections with the ACC and the amygdala (Figure 4), these trends were just below an alpha level of 0.05; that is, the probability values were *p* = 0.059 and *p* = 0.052, respectively. Indirect connections from the STP were also significantly greater in the water condition with the insula (*p* = 0.021), the ACC (*p* = 0.017), the hippocampus (*p* = 0.027), and marginally significant with the amygdala (*p* = 0.051).

Figure 5 illustrates the number of connections from the anterior cingulate cortex (ACC). The ACC had significantly more direct connections with the insula (*p* = 0.021) in the water condition and marginally significant greater direct connections with the STP (*p* = 0.059). However, the ACC had significantly more indirect connections to all four nodes in the water vs. the MR condition: insula (*p* = 0.009), STP (*p* = 0.024), amygdala (*p* = 0.041), and the hippocampus (*p* = 0.035).

Figure 6 illustrates the number of connections that the amygdala had within the water vs. MR condition. Direct connections were significantly greater with the insula (*p* = 0.045) with trends for the STP (*p* = 0.052) and hippocampus (*p* = 0.062). The amygdala also had a greater number of indirect connections with the insula (*p* = 0.016), with the STP (*p* = 0.022), and with the hippocampus (*p* = 0.015) in the water condition, whereas the effect with the ACC was marginally significant (*p* = 0.053).

**Figure 7:**
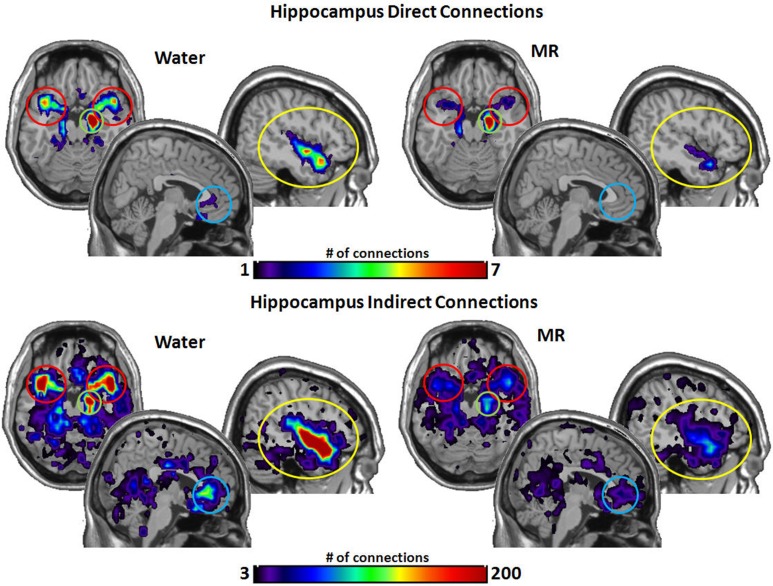
**The brain images on the top demonstrate the total number of direct connections from the right hippocampus averaged across participants**. *The top images demonstrate the right hippocampus has a greater number of direct connections with the insula and to the STP in the water condition than in the MR condition.* The brain images on the bottom demonstrate the total number of indirect connections from the right hippocampus averaged across individuals. *These images demonstrate that the right hippocampus has a greater number of indirect connections to the STP, ACC, and the insula during the water condition than the MR condition.*

Figure 7 captures the direct and indirect connections from the hippocampus. The hippocampus had a greater number of direct connections in the water condition with the insula (*p* = 0.029) and STP (*p* = 0.016). Indirect connections from the hippocampus were higher in the water condition with the insula (*p* = 0.022), the STP (*p* = 0.025), and the ACC (*p* = 0.048).

**Figure 8:**
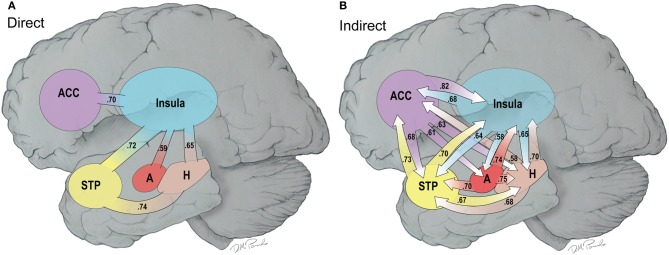
**(A,B)** This is a cartoon summary of the direct **(A)** and indirect **(B)** connections among the hubs of the HNB-A that are greater in the water vs. the MR condition. The connections are weighed by effect sizes between the two conditions which are shown as part of the arrows.

Figure 8 provides a composite cartoon of both the direct and indirect connections between the nodes of the HBN-A that were greater in the water than the MR condition. Because the direct connections are first order, we refer to this circuit as the primary circuit of the HBN-A, where we use the term secondary circuit to capture the second order or indirect connections. Effect sizes are provided for each connection with both the primary and secondary circuits to provide the reader with a sense of their relative strength. For the direct connections, the effect sizes ranged from 0.38 to 0.74; whereas, for indirect connections, the effect sizes ranged from 0.53 to 0.82. It is important to note that nearly all of these effect sizes are moderate to large in magnitude.

When examining the primary circuit of the HBN-A (Figure 8A), there are several notable features. First, the insula is a hub in the primary circuit as it has direct connections to every other node in the HBN-A. Second, there is also a sub-circuit which includes the insula, the hippocampus, and the STP (Figure 8A). And third, this sub-circuit was reproduced within the secondary circuit which involved indirect connections.

Whereas the structure of the primary circuit was found to be embedded within the secondary circuit, there were many more indirect connections between the nodes of the HBN-A. The indirect connections or the secondary circuit showed high connectivity between all 4 nodes with the exception of a few connections with the amygdala. Of note, the secondary circuit revealed a greater number of indirect connections with the ACC and the amygdala with the other regions in the HBN-A. Specifically, the ACC had outgoing, indirect connections to every other node in the HBN-A. The amygdala has greater outgoing, indirect connections to the insula, the hippocampus, and the STP. The amygdala was the only ROI in the HBN-A that did not have reciprocal indirect connections to every other ROI in the HBN-A. However, it is important to point out that the amygdala showed a trend of significantly greater indirect connections to both the ACC (*p* = 0.053) and the STP (*p* = 0.051).

## Discussion

The consumption of a meal replacement (MR) beverage in a group of older, obese adults after a short-term period of food restraint lowered state cravings and hunger ratings. As we shall see, these shifts observed with the MR were most likely due to the alleviation of the “hot-state” created by the short-term restriction from food. Specifically, consistent with these self-report data, we also demonstrated that consumption of a MR blunted connectivity in the hot-state brain network of appetite (HBN-A). To our knowledge, this is the first paper to show that a MR can modulate brain networks after a short-term period of food restraint. A novel feature is that using graph theory we identified a complex network of indirect connectivity within the HBN-A.

To define the HBN-A, we examined the resting brain network of the experimental trial in which individuals continued to refrain from eating; i.e., the water condition. Qualitatively, we observed that their brain networks had hubs in regions associated with the identification, processing, and the emotional elaboration of visceral food cues. These brain regions were defined as hubs-of-interest because they had a greater number of connections (degree) in the water vs. the MR condition. Once we defined these hubs, we were able to investigate the direct and indirect connections among these hubs between the two treatment conditions to fully characterize the network.

To our knowledge, this is the first study that uses graph theory to build and to study resting brain networks after a short-term period of food restraint. Graph theory is becoming an increasingly popular technique among neuroscientists to study human brain networks using fMRI; however, this technique is radically different from traditional fMRI methodologies as it allows the study of whole brain relationships. Thus, it is difficult to fully integrate this work with previous neuroimaging studies as we are most likely capturing a different network. As a result, we have decided to fully characterize this network and to name it the HBN-A.

In our investigation of the HBN-A, we found that when individuals continued to consume only water, their brain networks had hubs in the following regions: insula, superior temporal pole, hippocampus, ACC, and amygdala (see Figure 2). These hubs became the nodes of the HBN-A, having a long history of being identified with visceral sensations and hedonic attribution/elaboration (Gautier et al., 2001; Labar et al., 2001; Rothemund et al., 2007). Importantly, we do not believe the hubs defined in the HBN-A are the only regions that are active in a “hot-state.” It is more than likely that these regions are the up-stream result of primary, sub-cortical networks that have been well characterized as being important for eating behavior and addiction in animal studies (Kringelbach et al., 2012; Panksepp and Biven, 2012; Stice et al., 2013). Moreover, in the current study, there was substantially greater connectivity between these nodes in the water than MR condition. As shown in Figure 8, nearly all of these connections had moderate to large effect sizes (Cohen, 1992).

The insula, the hub of the primary circuit (Figure 8A), is well-known for its association with gustation and visceral sensations in general (Critchley, 2004; Kringelbach et al., 2012; Uddin et al., 2013); however, it has also been implicated in the experience of emotions derived from bodily states (Rothemund et al., 2007; Kringelbach et al., 2012). The insula is known to have connections with the superior temporal gyrus, the temporal pole, amygdala, hippocampus and the ACC (Shelley and Trimble, 2004; Nagai et al., 2007) which was evident in our data. The centrality of the insula to the HBN-A suggests that this network is viscerally-driven and is truly embodied. In other words, visceral cues from peripheral sensatory systems literally drive activity in this network (Rejeski and Gauvin, 2013). To confirm the visceral foundation of the HBN-A, future research using this paradigm should consider evaluating hormones known to be related to appetite and monitor potential changes in the autonomic nervous system.

The temporal pole (TP), the second node of the HBN-A, is considered a paralimbic region that is important for the multisensory processing of auditory, olfactory and visual stimuli (Olson et al., 2007). It has also been implicated in the emotional processing of these multisensory stimuli, and some studies have even argued that the anterior temporal lobe is important for emotional stability (Olson et al., 2007). The temporal pole is not a region that we originally hypothesized to be a hub of the HBN-A; however, it is not surprising since the TP is an important brain region for multisensory and emotional processing.

The hippocampus, another limbic structure and the third node of the HBN-A, is classically known to be important in memory; however, it has also been implicated in eating behavior (Tracy et al., 2001; Squire, 2004; Davidson et al., 2007; Bragulat et al., 2010). There is increasing evidence that the hippocampus is important for hedonic and incentive processes and for sensing the metabolic and hormonal status of the body (Lathe, 2001; Davidson et al., 2007; Bragulat et al., 2010). It is important to note that, in our analysis of the hippocampus, connectivity differences were unilateral and localized to the right-side. Interestingly, a study by Stoeckel et al. (2009) found that obese women have significantly greater unilateral activation in the right hippocampus in response to high-calorie foods compared to controls; of note, the obese women also had greater bilateral activations in the amygdala, insula, ACC, and several other brain regions as well.

The fourth node of the HBN-A is the ACC. The ACC has traditionally been associated with regulation of attention to both cognitive (dorsal portion) and emotional processing (ventral portion) and with goal-directed behavior (Devinsky et al., 1995; Bush et al., 2000; Mohanty et al., 2007; Gasquoine, 2013). The ventral (genual or anterior) region of the ACC, the region that was found to be part of the HBN-A in this study (see Figure 5), has been shown to be important for emotional processing (Lindgren et al., 2012; Gasquoine, 2013). Specifically it is important for evaluating and encoding the emotional salience (pleasantness/averseness) of various stimuli including the pleasantness of fat-content in drinks and human touch (Grabenhorst et al., 2010; Lindgren et al., 2012; Gasquoine, 2013). The ACC is capable of translating its emotional processing into a behavioral consequence, in part, via the autonomic nervous system (ANS) working in conjunction with the insula (Devinsky et al., 1995; Critchley et al., 2003; Gasquoine, 2013).

The fifth and final node of the HBN-A was the amygdala. The amygdala's role in fear and aversion is well known; however, the amygdala is now considered to be important for valence attribution, as it responds to both aversive and appetitive stimuli (Ball et al., 2009). The amygdala has connections to the insula in humans (Shelley and Trimble, 2004) and to the temporal pole in marquee monkeys (Olson et al., 2007), connections we observed in our data.

The primary circuit of the HBN-A (Figure 8A) has a sub-circuit that includes the insula, the hippocampus and the STP. This sub-circuit has the capacity to integrate visceral sensation from the insula with memory/metabolic status from the hippocampus and emotional processing of multisensory stimuli from the STP. In addition, this sub-circuit has access to emotional information from the ACC and the amygdala through the hub of the insula.

Importantly, a novel feature of this study is our ability to delineate indirect connections and thereby to create a secondary circuit. With traditional neuroimaging techniques, this would have been impossible and only the primary circuit (i.e., direct connections) could have been elucidated. However, by using graph theory to define our brain network, we were able to analyze indirect connections leading to the identification of the secondary circuit. This secondary circuit underscores how highly interconnected the ACC and the amygdalae are with the other regions in the HBN-A in the water condition (see Figure 8B). Using the direct connections only, it would appear that the ACC and the amygdalae must filter their information into the primary circuit through the hub of the insula; however, the identification of the secondary circuit suggests that this is an oversimplification. Many of the indirect connections from the ACC and amygdalae are through the insula; however, the robustness of their indirect connectivity could not have been predicted from the direct connections alone.

The secondary circuit illustrates that the indirect connections among the hubs of the HBN-A are reciprocally related to one another and highly distributed. This gives credence to the idea that brain networks are complex and do not function in a linear manner. Thus, the HBN-A is truly a circuit where sensory stimuli [visceral (insula); multisensory (STP)] and memory/metabolic status (hippocampus) are directly integrated with one another and further elaborated upon emotionally via the secondary circuit involving the ACC and the amygdala. Therefore, graph theory provides novel and important information to our understanding of functional brain network circuitry and should be further explored in future applications of functional connectivity.

Generally speaking, most regions in the HBN-A operate below the level of consciousness; thus, when people are in a “hot state” it is likely that behavior is controlled to a significant degree by automatic processes. Loewenstein (2005) has shown that when people are in “cold states” they overestimate their self-regulatory capacities; in other words, they might believe that they can control their eating behavior immediately after a noon meal, yet fail miserably at controlling consumption by mid-afternoon. Thus, future research should examine the role of the HBN-A on one's ability to control consumption. It is important to note that since MR beverages are low scoring hedonic products and are often less liked than “regular” food, it is possible that other regions may be important during active attempts at self-regulation. For instance, after a short term fast, a tasty food product may not only trigger the hubs identified in this study, but also portions of the hedonic system such as the OFC creating a more complex network. In short, at this point in time, we do not know whether the structure of the HBN-A or connectivity within this network is generalizable beyond the realm of meal replacements.

This current study is not without limitations. First, the small sample size makes us sufficiently underpowered to investigate individual differences in the HBN-A. Secondly, the target sample was restricted to an older, overweight and obese population that was not currently dieting. It is possible that differences in brain networks may have been observed if participants had been actively engaged in intentional weight loss. We also did not have a normal weight control group, meaning that these results may be limited to older adults that are overweight or obese. The results of this study should be considered specific to the population under investigation until subsequent studies confirm these effects in younger age-groups and people with more diverse biometric characteristics. Finally, the reader should be aware that we used a *per comparison* error rate for evaluating the individual connections between hubs of interest and supplemented these analyses with effect sizes. Some may view this approach as too liberal and likely to create type I errors. On the other hand, the small sample sizes often used in imaging studies makes correction for multiple comparisons challenging. We want to underscore the moderate to strong effect sizes for all comparisons conducted between the water and MR conditions providing consistent support for the HBN-A using cutting edge methods from graph theory.

In summary, our qualitative and data-drive approach was successful in defining a well interconnected HBN-A. The plausibility of the HBN-A network is supported by its significant attenuation during the consumption of a MR as compared to water. This network included many regions previous implicated in eating behavior and describes a viscerally-driven network that is rich in valence attribution and incentive-motivated processing. Furthermore, the findings of this study demonstrate that MR beverages are able to down-regulate the HBN-A, and reduce food craving/hunger following a short-term period of food restraint in older, overweight and obese adults as compared to water. In light of these findings, further research is warranted to examine how the HBN-A is related to peoples' ability to stick with daily caloric goals typically set in weight management programs. In other words, is the HBN-A a brain signature for self-regulatory failure when attempting weight loss? Does the HBN-A have important relationships with other brain regions, such as the OFC, during active attempts at self-regulation? We are beginning to address some of these questions. Currently, we are examining whether differences in the HBN-A in response to an overnight fast predicts weight loss behavior during an active weight loss intervention. Additionally, we will also investigate if weight loss blunts the HBN-A response post intervention.

### Conflict of interest statement

The authors declare that the research was conducted in the absence of any commercial or financial relationships that could be construed as a potential conflict of interest.
